# Relative contributions of endogenous and exogenous formaldehyde to formation of deoxyguanosine monoadducts and DNA-protein crosslink adducts of DNA in rat nasal mucosa

**DOI:** 10.1093/toxsci/kfac119

**Published:** 2022-11-21

**Authors:** Rory B Conolly, Jerry L Campbell, Harvey J Clewell, Jeffry Schroeter, Julia S Kimbell, P Robinan Gentry

**Affiliations:** Ramboll US Consulting, Inc., Monroe, Louisiana 71201, USA; Ramboll US Consulting, Inc., Monroe, Louisiana 71201, USA; Ramboll US Consulting, Inc., Monroe, Louisiana 71201, USA; Applied Research Associates, Inc., Raleigh, North Carolina 27615, USA; Orleans, Massachusetts 02653, USA; Ramboll US Consulting, Inc., Monroe, Louisiana 71201, USA

**Keywords:** formaldehyde, DNA adducts, endogenous, nasal, dosimetry, CFD, BBDR

## Abstract

Understanding the dose-response for formaldehyde-induced nasal cancer in rats is complicated by (1) the uneven distribution of inhaled formaldehyde across the interior surface of the nasal cavity and, (2) the presence of endogenous formaldehyde (endoF) in the nasal mucosa. In this work, we used computational fluid dynamics (CFD) modeling to predict flux of inhaled (exogenous) formaldehyde (exogF) from air into tissue at the specific locations where DNA adducts were measured. Experimental work has identified DNA-protein crosslink (DPX) adducts due to exogF and deoxyguanosine (DG) adducts due to both exogF and endoF. These adducts can be considered biomarkers of exposure for effects of endoF and exogF on DNA that may be part of the mechanism of tumor formation. We describe a computational model linking CFD-predicted flux of formaldehyde from air into tissue, and the intracellular production of endoF, with the formation of DPX and DG adducts. We assumed that, like exogF, endoF can produce DPX. The model accurately reproduces exogDPX, exogDG, and endoDG data after inhalation from 0.7 to 15 ppm. The dose-dependent concentrations of exogDPX and exogDG are predicted to exceed the concentrations of their endogenous counterparts at about 2 and 6 ppm exogF, respectively. At all concentrations examined, the concentrations of endoDPX and exogDPX were predicted to be at least 10-fold higher than that of their DG counterparts. The modeled dose-dependent concentrations of these adducts are suitable to be used together with data on the dose-dependence of cell proliferation to conduct quantitative modeling of formaldehyde-induced rat nasal carcinogenicity.

Although exposure to exogenous formaldehyde (exogF) is associated with nasal cancer in the F344 rat at cytotoxic concentrations, only equivocal epidemiological evidence of cancer due to inhalation of formaldehyde has been found for humans. The observation of nasal cancer in F344 rats is well supported by extensive mechanistic studies (summarized by Thompson *et al*. (2020)), whereas the evidence for human nasal cancer is mixed (Andersen *et al*., 2019; Beane Freeman *et al*., 2013; Blair *et al*., 1986; Coggon *et al*., 2014; Hauptmann *et al*., 2004; Marsh *et al*., 2007; Pinkerton *et al*., 2004). Because of this qualitative difference between the rat and human datasets, it has been of long-standing interest to understand the implications for human risk of the mechanistically rich rat dataset. The relevance of better understanding this difference is highlighted by the recent release from the U.S. EPA of a new draft cancer risk assessment for formaldehyde (USEPA, 2022). This draft assessment includes extensive coverage of the mechanistic studies, which are primarily in rats, and of the biologically based dose-response (BBDR) modeling by Conolly *et al*. (2003, 2004), but ultimately relies on epidemiological studies to identify acceptable levels of exposure.

Conolly *et al*. (2003) described a BBDR model for the nasal carcinogenicity of inhaled formaldehyde in the F344 rat and Conolly *et al*. (2004) described the scale-up of this model to humans. The rat BBDR model consists of (1) Computational fluid dynamics (CFD) modeling describing the relationship between the inhaled concentration of formaldehyde and the site-specific flux of formaldehyde from air within the nasal cavity into the mucosal tissue lining the cavity (Kimbell *et al*., 1997, 2001b), (2) a tissue dosimetry model that links CFD-predicted flux of formaldehyde into nasal mucosal tissue with the formation of DNA-protein crosslinks (DPX) (Conolly *et al*., 2000), and (3) a 2-stage clonal growth model for cancer that combines the predicted DPX burden and other relevant mechanistic data as inputs to predict the nasal tumor dose-response and time-course. However, since the Conolly *et al*. (2003, 2004) manuscripts were published, CFD methodology has been refined and new data on deoxyguanosine (DG) DNA monoadducts formed following formaldehyde inhalation exposure have been described (Leng *et al*., 2019; Lu *et al*., 2011; Yu *et al*., 2015). Of particular interest from the DG adduct work is the identification of adducts due to endogenous formaldehyde (endoF), which occurs naturally in cells as part of 1-carbon metabolism. The older DPX data are only available for exogF. The refinements in CFD methodology and the availability of data on DNA adducts due to endoF opens the door for a revision of both the DNA adduct and the BBDR modeling.

Campbell *et al*. (2020) described a tissue dosimetry model for DG adducts due to both exogF and endoF but did not incorporate CFD-derived, site-specific predictions of flux from air into tissue, nor the DPX data, into their analysis. Here, we describe the technical refinements in the CFD modeling of exogF in the F344 rat nasal cavity, use of the CFD modeling to predict fluxes of exogF into nasal mucosal tissue at the specific tissue sites where DPX and DG adducts were measured, and the addition of DPX to the adduct modeling. This new CFD/tissue dosimetry model predicts F344 rat nasal cavity mucosal tissue levels of endogenous DG and DPX (endoDG; endoDPX) and exogenous DG and DPX (exogDG; exogDPX) and supports the characterization of flux bins that provide a high-resolution description of how flux of formaldehyde from air into tissue varies throughout the rat nasal cavity (Kimbell *et al*., 2001a). These predictive capabilities, while of intrinsic interest for the insights they provide into the relative roles of endoF and exogF in the formation of DG and DPX adducts, are needed for an ongoing update of the BBDR modeling of formaldehyde carcinogenicity in the F344 rat nose that was described by Conolly *et al*. (2003). Extrapolation of the model to humans can then inform us about the potential for human risk of the tumors seen in rats and, potentially, provide insights into the quantitative, mechanistic requirements for the development of human respiratory tract tumors associated with exogF. The revised BBDR modeling will be described in a separate publication.

## Materials and methods

###  

####  

##### Overview

Prediction of nasal mucosal levels of DG and DPX adducts, due to both endoF and exogF, requires (1) CFD modeling that translates the inhaled concentration of formaldehyde into flux of formaldehyde into tissue at the specific sites where adducts were measured, and (2) tissue dosimetry modeling that integrates the site-specific flux with the description of mucosal kinetics of formaldehyde and formation of DG and DPX adducts.

##### CFD modeling

**Figure 1.**
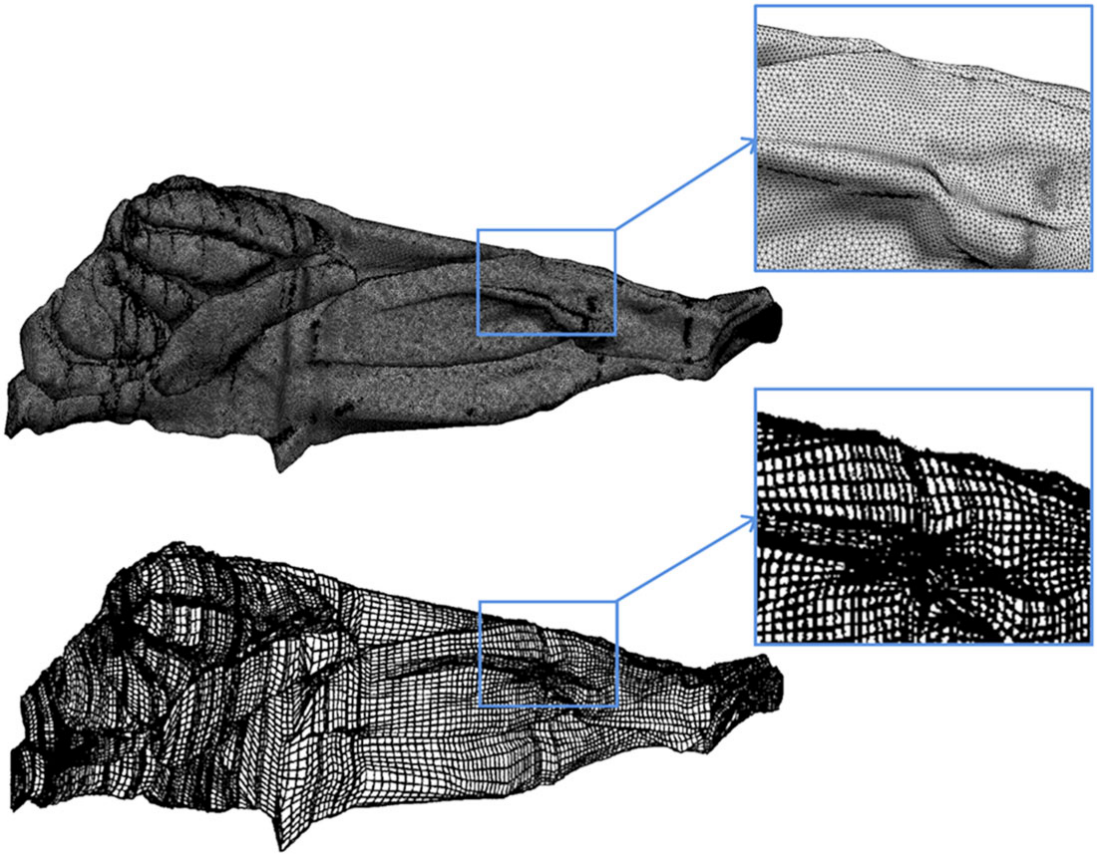
Computational mesh comparison. Views are of the right side of the nose, with the nostrils to the right, showing the tetrahedral mesh of the smoothed F344 rat model (top panels) used in the present work, and the hexahedral mesh of the original F344 rat model (bottom panels) used in the previous work (modified from Fig. 1 in Kimbell *et al*. (1997), used with permission). Insets show mesh close-ups for more detailed comparison.

**Figure 2.**
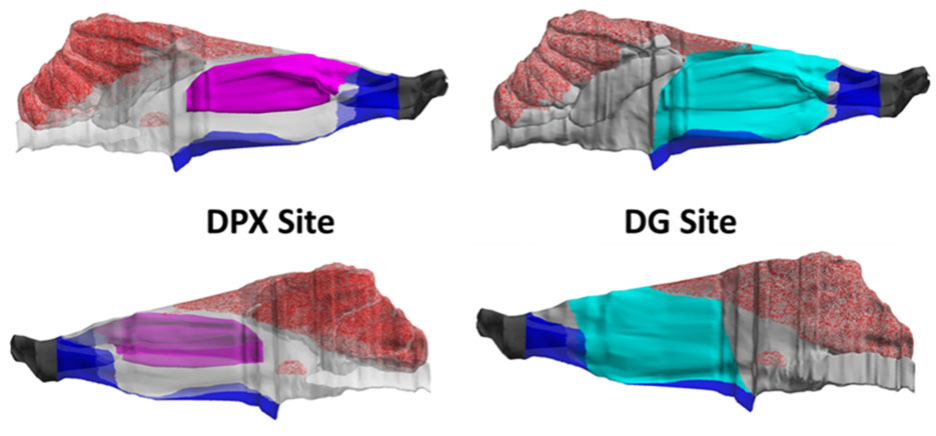
Lateral (top panels) and septal (bottom panels) views of the F344 rat nasal CFD model with the locations of the high tumor DPX site (left, magenta) and DG site (right, light blue) mapped onto the model. Epithelial types included in the surface model include dry squamous (dark grey), wet squamous (dark blue), respiratory/transitional (light grey), and olfactory (red cross-hatch). The DPX site was described by Casanova *et al*. (1994) and did not include any septal tissue; the DG site was defined based on descriptions by Lu *et al*. (2011) and Doyle-Eisele (2020) and consisted of nasal respiratory epithelium on the lateral and septal walls. The DG site encompassed a larger surface area that included regions outside of the main flow streams and therefore resulted in slightly lower average flux compared to the DPX site. Abbreviations: CFD, computational fluid dynamics; DG, deoxyguanosine; DPX, DNA protein cross-link.

The rat nasal dosimetry of formaldehyde is not uniform, having a steep, decreasing, anterior to posterior gradient of flux into tissue (Kimbell *et al*., 1997). The ability to use CFD modeling to predict flux of formaldehyde from inhaled air into specific sites in the nasal cavity, and thereby reproduce the anterior-posterior flux gradient, was provided by an anatomically accurate, 3-dimensional (3D) reconstruction of the nasal passages developed for an adult male F344 rat (Schroeter *et al*., 2014; Figure 1). Most of formaldehyde-induced rat nasal tumors occur in the anterior nasal cavity “high tumor” site (Figure 2) as specified by Casanova *et al*. (1994). The high tumor site was mapped onto the CFD model as described by Kimbell *et al*. (2001b) (Figure 2). DG adducts were measured in a site that includes the high tumor region and, in addition, the medial aspects of the naso- and maxilloturbinates as well as the adjacent septum (Thompson *et al*., 2020; Doyle-Eisele 2020; Figure 2). These high tumor and DG adduct mappings enable the CFD model to predict average flux into tissue for the specific sites where DPX and DG adducts were measured.

Numerical meshes and steady-state inhalation airflow and formaldehyde transport simulations were created using the commercial software ICEM-CFD and Fluent (ANSYS, Inc, Canonsburg, Pennsylvania) as described in detail elsewhere (Schroeter *et al*., 2014), except that the boundary conditions used for formaldehyde transport were described by Kimbell *et al*. (2001b). Simulation of the full breathing cycle, rather than of steady-state inspiratory flow, is technically feasible (Corley *et al*., 2015). However, we used steady-state inspiratory airflow as it is computationally faster and as noted by Corley *et al*. (2015), provides comparable predictions of peak and average concentration in the rat nasal cavity for reactive aldehydes such as formaldehyde. Although Corley *et al*. (2015) observed differences in area under the curve predictions between steady-state and transient simulations, transient calculations were computed over an entire breath including inhalation and exhalation. Due to the nearly complete absorption of inhaled formaldehyde in the respiratory tract upon inhalation, use of steady-state flux calculations that are representative of average flux over the inhalation period is appropriate. User-defined functions were written and compiled into Fluent to compute the rate at which formaldehyde was absorbed by airway walls regionally (average formaldehyde flux) by summing mass flows (mass/time) on all elements in the region and dividing by the surface area of that region.

##### DNA adduct data

A combined dataset for monoadducts of DNA and DPX was assembled from the DG monoadduct data used by Campbell *et al*. (2020), the DPX crosslink data used by Conolly *et al*. (2000), and additional crosslink data described by Lai *et al*. (2016) and Leng *et al*. (2019) (Table 1). These latter 2 studies measured crosslinks specifically at deoxyguanosine (DPC), which are a subset of crosslinks at all 4 DNA bases. The DPX data (Casanova *et al*., 1989, 1994) were obtained using a less specific method that detects crosslinks (DPX) at all 4 bases. Because, with exposure to either 2 or 15 ppm formaldehyde, measured exogDPC are several orders of magnitude lower in concentration than exogDPX (Table 2), we focused our modeling efforts on the exogDPX and the DG monoadduct datasets.

**Table 1.** Deoxyguanosine adduct and DNA-protein crosslink data

Source	Formaldehyde (ppm)	Time^d^ (h)	Monoadducts		DNA-protein crosslinks		
			endoDG^e^ (pmol/mm^3^)	exogDG^e^ (pmol/mm^3^)	endoDPC (pmol/mm^3^)	exogDPC (pmol/mm^3^)	exogDPX (pmol/mm^3^)
Lu *et al*. (2011) ^a^	0.7	7	9.527e−4	1.026e−5	—	—	—
	2.0	7	1.603e−3	5.001e−5	—	—	—
	5.8	7	1.450e−3	2.737e−4	—	—	—
	9.1	7	8.975e−4	5.343e−4	—	—	—
	15.2	7	1.116e−3	2.935e−3	—	—	—
Yu *et al*. (2015) ^b^	2.0	175	6.606e−4	9.212e−5	—	—	—
	2.0	343	8.132e−4	2.211e−4	—	—	—
	2.0	511	8.790e−4	2.500e−4	—	—	—
	2.0	679	7.422e−4	2.763e−4	—	—	—
	2.0	684	7.369e−4	2.184e−4	—	—	—
	2.0	702	7.843e−4	2.106e−4	—	—	—
	2.0	750	7.869e−4	1.658e−4	—	—	—
	2.0	846	7.317e−4	1.763e−4	—	—	—
Lai *et al*. (2016)	control		—	—	1.711e−5	—	—
	2.0	178	—	—	1.258e−5	2.527e−6	—
	2.0	192	—	—	1.187e−5	6.474e−6	—
	2.0	696	—	—	9.948e−6	5.580e−6	—
	2.0	840	—	—	9.238e−6	5.632e−6	—
	15.0	24	—	—	1.163e−5	1.453e−5	—
	15.0	48	—	—	1.126e−5	1.234e−5	—
	15.0	96	—	—	9.659e−6	4.785e−5	—
Leng *et al*. (2019)	control		—	—	7.001e−6	—	—
	0.001	672	—	—	7.290e−6	not detected	—
	0.030	672	—	—	7.922e−6	not detected	—
	0.300	672	—	—	7.501e−6	not detected	—
Casanova *et al*. (1989)	0.32	6	—	—	—	—	5.74e−3
Casanova *et al*. (1994) ^c^	0.72	1947	—	—	—	—	9.02e−3
	2.06	1947	—	—	—	—	2.42e−2
	6.01	1947	—	—	—	—	1.94e−1
	15.8	1947	—	—	—	—	1.14

a Rats were exposed once for 6 h and were reported as sacrificed within 2 h following exposure. We chose 1 h post-exposure, or 7 h from the start of the exposure, for *in silico* modeling.

b Rats were exposed to 2 ppm, for 6 h/day, for 7, 14, 21, or 28 consecutive days and were reported as sacrificed within 2 h of the end of exposure. We chose 1 h post-exposure, or 7 h from the start of the daily exposure, for *in silico* modeling. After the 28th exposure, animals were sacrificed at 6, 24, 72, and 168 h post-exposure.

c Rats were exposed 6 h/day, 5 days/week for 11 weeks and 4 days. On the 5th day of the 12th week, rats were exposed for 3 h, then sacrificed immediately.

d Time from the start of the first exposure to collection of adduct data.

e DG monoadduct data were reported in units of adducts/10^7^ deoxyguanosine, whereas the DPC crosslink data were reported in units of crosslinks/10^8^ deoxyguanosine. These were transformed to pmol adduct/mm^3^ tissue for comparability with the exogDPX data.

**Table 2.** Parameter values

Parameter	Process described	Value	Units	Source
kp	Endogenous production of formaldehyde	10 835	pmol/mm^3^/h	Optimized
k21	First-order loss of formaldehyde	16.57	1/h	Optimized
k23	Second-order constant for formation of formaldehyde-GSH complex	0.35	1/pmol/mm^3^/h	Optimized
K32	First-order dissociation of formaldehyde-GSH complex	200	1/h	Campbell *et al*. (2020)
kDNA_DG	First-order formation of DG adducts	9.5183e−8	1/h	Optimized
kDNA_DPX	First-order formation of DPX adducts	1.8183e−4	1/h	Optimized
krep_DG	First-order loss DG adducts	0.0063	1/h	Campbell *et al*. (2020)
krep_DPX	First-order loss DPX adducts	0.39	1/h	Conolly *et al*. (2000)
Vmax	Maximum rate of oxidation of formaldehyde-GSH complex by formaldehyde dehydrogenase	74156	pmol/mm^3^/h	Optimized, near Conolly *et al*. (2000)
Km	Affinity constant for formaldehyde-GSH complex with formaldehyde dehydrogenase	1935	pmol/mm^3^	Optimized
thick	Mucosal thickness	0.1138	mm	Optimized
endoF_init	Initial concentration endoF	20.0	pmol/mm^3^	Campbell *et al*. (2020) ^a^
GSH_init	Initial concentration GSH	4506	pmol/mm^3^	Optimized
endoF_GSH_init	Initial concentration endoF_GSH complex	120.	pmol/mm^3^	Campbell *et al*. (2020) ^a^
endoDG_init	Initial concentration endoDG	7.42e−4	pmol/mm^3^	Optimized against Yu *et al*. (2015)
endoDPX_init	Initial concentration endoDPX	0.01	pmol/mm^3^	Set manually^a^
flux_DPX	Flux into DPX high tumor sampling site	800.3	pmol/mm^2^/h	CFD prediction
flux_DG	Flux into DG sampling site	776.6	pmol/mm^2^/h	CFD prediction

a Multi-day simulation are not sensitive to this value.

The DG adduct data (Lu *et al*., 2011; Yu *et al*., 2015) were described with units of adducts/10^7^ deoxyguanosine and the DPC crosslink data (Lai *et al*., 2016; Leng *et al*., 2019) with units of adducts/10^8^ deoxyguanosine, while the DPX data (Casanova *et al*., 1989, 1994) have units of pmol/mm^3^. The current work required that all adduct data have the same units. For this reason, the DG and DPC data were converted to pmol/mm^3^. This conversion is described in Appendix 1. All the adduct data, with common units, are provided in Table 1.

##### Tissue dosimetry modeling

**Figure 3.**
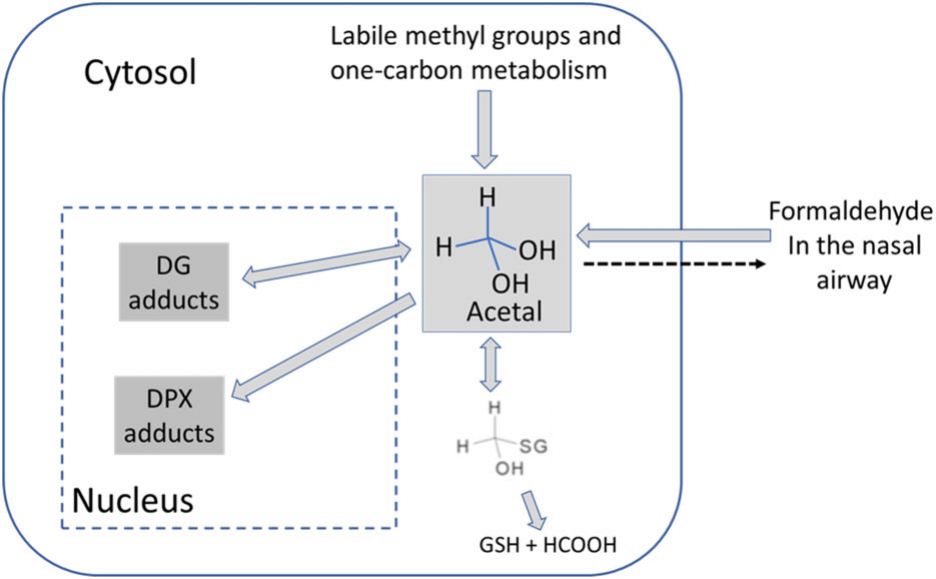
Formation of deoxyguanosine (DG) and DNA-protein crosslink (DPX) adducts in the rat nasal mucosa. Labile methyl groups and 1-carbon metabolism are responsible for the endogenous production of formaldehyde, whereas exogenous formaldehyde is absorbed from the nasal airway. Intracellular formaldehyde exists largely as its hydrate, formaldehyde acetal. In the nucleus, formaldehyde reacts with DNA to form DG and DPX adducts.

The present work extends the tissue dosimetry model of Campbell *et al*. (2020) in 2 ways—the use of CFD modeling to describe site-specific tissue uptake of formaldehyde from air and the inclusion of DPX adducts (Figure 3).

Campbell *et al*. (2020) calculated uptake, the rate at which formaldehyde flows from air into tissue, by assuming that the flux of inhaled formaldehyde into tissue was the same across the entire nasal epithelial surface area. This approach did not require use of CFD modeling and did not allow for prediction of site-specific flux and uptake, as is needed for use of CFD dosimetry predictions in the current work and in BBDR modeling (Conolly *et al*., 2003).

Calculation of site-specific uptake has the form:

(1) $$\mathrm{uptake}=\;\left(\frac{\mathrm{pmol}}{{mm}^{2}-hr-\mathrm{ppmf}}\right)\times \left(\frac{1}{\mathrm{thick}}\right)\times \mathrm{ppm}$$

where *uptake* (pmol/mm^3^/h) is a function of site-specific *flux* (pmol/mm^2^/h/ppmf) predicted by CFD modeling for inhaled concentration *ppmf*, *thick*, the thickness of the mucosal tissue (mm), and inhaled *ppm* formaldehyde. Flux is predicted for *ppmf *=* *1 and is assumed to be linear with the inhaled concentration *ppm*. This approach allows for specification of specific fluxes for the sites at which DPX and DG adducts were measured and for the use of flux binning in BBDR modeling (Conolly *et al*., 2003; Kimbell *et al*., 2001a).

DPX are calculated separately for formaldehyde of endogenous and exogenous origin:

(2) $$\frac{\mathrm{dendoDPX}}{dt}=k_{\mathrm{DNA}\_\mathrm{DPX}}\;\times \;\left[\mathrm{endoF}\right]-k_{\mathrm{rep}\_\mathrm{DPX}}\times \;\left[\mathrm{endoDPX}\right]$$

where *k_DNA_DPX_* is the first-order constant for formation of DPX adducts and $k_{\mathrm{rep}\_\mathrm{DPX}}$ is the first-order constant (1/h) for the repair or clearance of DPX. The same form is used in the equation for exogDPX:

(3) $$\frac{\mathrm{dexogDPX}}{dt}=k_{\mathrm{DNA}\_\mathrm{DPX}}\;\times \;\left[\mathrm{exogF}\right]-k_{\mathrm{rep}\_\mathrm{DPX}}\times \;\left[\mathrm{exogDPX}\right]$$

Addition of DPX to the dosimetry model also alters the differential equation for endoF in the nasal mucosa:

(4) $$\frac{\mathrm{dendoF}}{dt}=kp-\;k_{21}\times \left[\mathrm{endoF}\right]-k_{23}\times \left[\mathrm{endoF}\right]\times \left[\mathrm{GSH}\right]+k_{32}\times \left[\mathrm{endoF}\_\mathrm{GSH}\right]-k_{\mathrm{DNA}\_DG}\times \left[\mathrm{endoF}\right]-k_{\mathrm{DNA}\_\mathrm{DPX}}\times \left[\mathrm{endoF}\right]$$

where *kp* is the zero-order rate of production of *endoF* (pmol/mm^3^/h), *k_21_* is the first-order rate constant for clearance or loss of *endoF* (1/h), *k_23_* is the 2nd-order constant for formation of the *endoF* complex with *GSH* (1/pmol/mm^3^/h), *k_DNA_DG_* is the first-order constant for DG adduct formation by *endoF* (1/h), and *k_DNA_DPX_* is the first-order constant for formation of DPX adducts (1/h). For exogF, the differential equation has the form:

(5) $$\frac{\mathrm{dexogF}}{dt}=\mathrm{uptake}-k_{21}\times \left[\mathrm{exogF}\right]-k_{23}\times \left[\mathrm{exogF}\right]\times \left[\mathrm{GSH}\right]+k_{32}\times \left[\mathrm{exogF}\_\mathrm{GSH}\right]-k_{\mathrm{DNA}\_DG}\times \left[\mathrm{exogF}\right]-k_{\mathrm{DNA}\_\mathrm{DPX}}\times \left[\mathrm{exogF}\right]$$

with parameters as defined above. The remaining differential equations of the tissue dosimetry model are as described by Campbell *et al*. (2020).

##### Parameter values

The models by Campbell *et al*. (2020) and Conolly *et al*. (2000) provided good descriptions of the DG and DPX adduct data, respectively. For the current work, parameters were initially given values from those works. However, the current model does differ in the description of formaldehyde uptake from air into tissue and in the simultaneous description of DG and DPX adducts (Figure 3). Adjustment of parameter values was initially conducted manually and then formally optimized, as described below. The final parameter values are a mix of values taken from the previous work and newly optimized values, as specified in Table 2.

##### Coding and optimization

Model code was written in Octave (Eaton *et al*., 2019). Optimization used fminsearchbnd© (D’Errico 2021), which allows specification of upper and lower bounds to constrain parameter optimization to biologically reasonable values. Fminsearchbnd interfaces with the fminsearch algorithm that is part of the Octave Optim package. Optim implements the Nelder-Mead simplex algorithm. These algorithms were used with a modified least squares cost function (Conolly *et al*., 2000; Steiner *et al*., 1990). Parameter values were optimized by minimizing the cost function against all the relevant data (Casanova *et al*., 1994; Lu *et al*., 2011; Yu *et al*., 2015) simultaneously. The model code can be found in the Supplementary material.

## Results

###  

#### CFD modeling

Simulated streamlines of inspiratory airflow with the current, refined CFD model (Schroeter *et al*., 2014; Figure 1) were qualitatively like the airflow results from Kimbell *et al*. (2001b). Mass balance errors for the airflow simulations were less than 0.007%.

Nasal uptake was predicted to be 87.4%, which is similar to the value of 90.0% reported by Kimbell *et al*. (2001b). The mass balance error for predicted uptake was <0.14%.

The estimated surface area of the site in which DG adducts were measured (419.9 mm^2^ for both sides of the nose) was almost 2.5-fold larger than the surface area of the high tumor region (171.6 mm^2^ for both sides of the nose). The additional tissues in the DG adduct site likely contribute to the somewhat lower average wall mass flux of formaldehyde predicted for this site (776.6 pmol/mm^2^-h) compared to the high tumor site (800.3 pmol/mm^2^-h). These flux values are averaged over one breath.

#### DNA adduct data

EndoDG and exogDG data were converted from their originally reported units of DG adducts/10^7^ DG to pmol DG adducts/mm^3^ tissue (Appendix 1; Table 1). Because the DG data are specific to deoxyguanosine, whereas the DPX data represent all DPX, one might expect that, for a given exposure, the DG data values would be about 33% of the DPX data values. However, DPX values are 10-fold or greater than the DG values (Table 1). This unexpected difference may be real or may reflect the different analytical methods used for the DPX and DG work (eg, Casanova *et al*., 1989; Lu *et al*., 2011), or some combination of these factors. This concern could be addressed by quantitative characterization of DNA adducts due to exogenous and endogenous formaldehyde using a consistent methodology.

**Figure 4.**
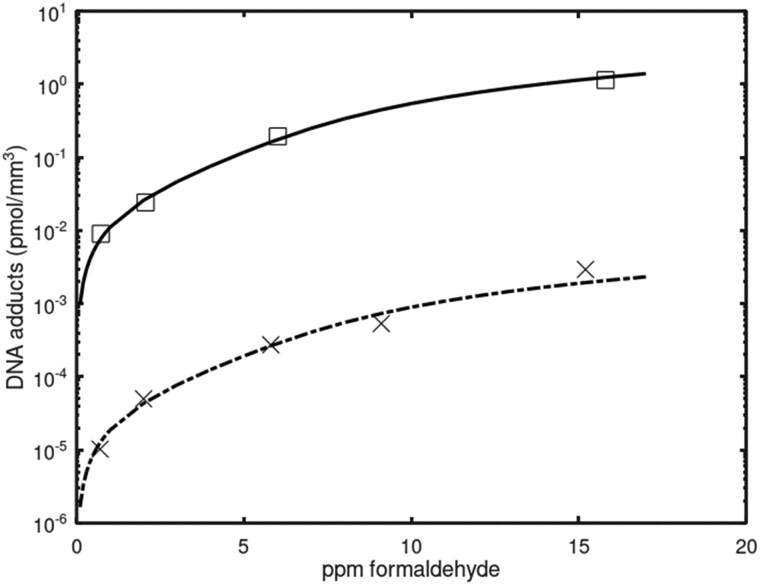
Dose-response simulations for DG (lower curve) and DPX (upper curve) adducts. The DG data are from Lu *et al*. (2011) (Table 1). The DPX data are from Casanova *et al*. (1994) (Table 1). DG adducts were measured at 7 hr from the start of a single 6-h exposure. DPX were measured immediately after a 3-h exposure that followed 11 weeks and 4 days of exposure, 6 h/day/days per week. Abbreviations: DG, deoxyguanosine; DPX, DNA-protein crosslink.

**Figure 5.**
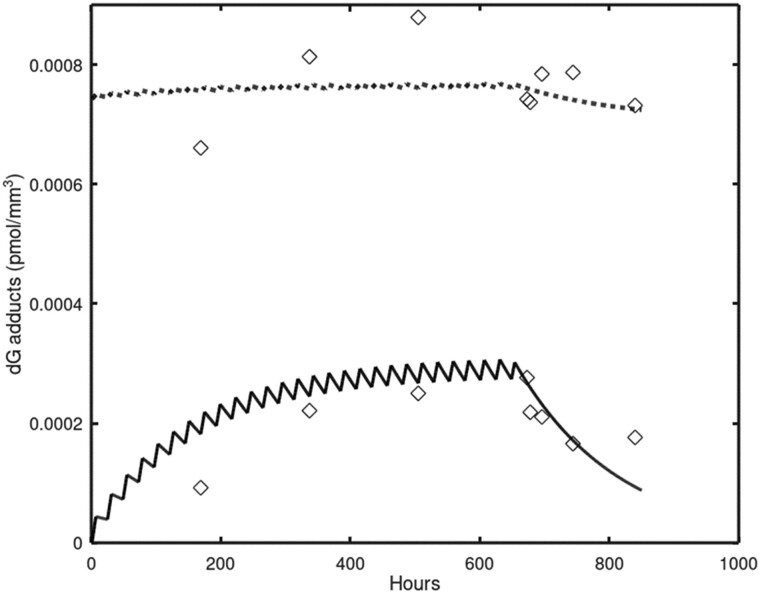
Simulation of the Yu *et al*. (2015) DG adduct data obtained during and after 28 days of exposure to 2.0 ppm formaldehyde. The lower, sawtooth curve is for exogenous DG adducts, whereas the upper curve is for endogenous DG adducts. Abbreviation: DG, deoxyguanosine.

Accurate descriptions of the adduct data were obtained (Figs. 4 and 5) using the parameters listed in Table 2. The measured exogenous adduct concentrations (Figure 4) extend over 4 orders of magnitude and were produced by exposures ranging from 0.7 to 15 ppm (Table 1). The time course DG adduct data were collected over 28 days of exposure at 2 ppm inhaled formaldehyde with further data collection for 7 days post-exposure. Although the exogenous DG adducts reach a steady-state level during the 28 days of exposure, their measured concentrations remain well below those of the endogenous DG adducts.

#### Parameter values

Many of the optimized parameter values (Table 2) were similar to values reported in Campbell *et al*. (2020) and Conolly *et al*. (2000). The zero-order rate of production of endoF increased from 4200 (Campbell *et al*., 2020) to 10 835 pmol/mm^3^/h, whereas the rate constant for first-order loss of formaldehyde was 16.57/h, about 3-fold greater than the value used by Campbell *et al*. (2020). The value of GSH_init, the initial concentration of GSH, increased from 2000 (Campbell) to 4506 pmol/mm^3^, or to about 4.5 mM. The value of k23, the 2nd-order rate constant for the interaction of endoF and exogF with GSH (0.35 1/pmol/mm^3^/h) was only slightly different from the value (0.30) used by Campbell *et al*. (2020), whereas the dissociation constant for the complex, k32, was identical to that used by Campbell *et al*. (2020).

The optimized value for Vmax was 74 156 pmol/mm^3^/h, slightly more than the 60 480 pmol/mm^3^/h used by Conolly *et al*. (2000) and about twice the value used by Campbell *et al*. (2020). The optimal value for km, 1935 pmol/mm^3^, is almost 5 times larger than the value used by Campbell *et al*. (2020) and much larger, almost 300-fold, than that used by Conolly *et al*. (2000). This set of parameter values (Table 2) does not suggest any clear-cut difference in the biological interpretation of the model from the work of Campbell *et al*. (2020).

The rate constants for formation of DPX and DG adducts were optimized, whereas the repair rate for DPX was taken from Conolly *et al*. (2000) and that for DG from Campbell *et al*. (2020). The higher concentration of DPX relative to DG adducts is due, in part, to the much larger rate constant for DPX formation (1.8183e−4/h) relative to that for DG formation (9.5183e−8/h). This difference outweighs the slower rate of repair of DG relative to DPX adducts. The fact that guanosine base pairs comprise only about 41% of the rat genome (Piovesan *et al*., 2019) also contributes to the difference in DPX and DG adduct concentrations.

Although there are regional differences in nasal distribution of formaldehyde dehydrogenase (Keller *et al*., 1990) and in epithelial thickness (Georgieva *et al*., 2003), the planned use of the CFD/adduct dosimetry model described here in support of BBDR modeling (eg, Conolly *et al*., 2003) requires that a uniform distribution of formaldehyde dehydrogenase activity and a single tissue thickness be used for the entire rat nasal mucosa. This constraint occurs because the spatial resolutions of the formaldehyde dehydrogenase activity and tissue thickness data are not comparable to the spatial resolution of the CFD-generated flux predictions used for flux binning (Kimbell *et al*., 2001a). Inclusion of data on regional variation in formaldehyde dehydrogenase activity and in tissue thickness is desirable but is beyond the capabilities of the currently available data. Therefore, model development proceeded in 2 stages. In the first stage (data not shown), the tissue thickness parameter (*thick*) was optimized separately against the DG and DPX adduct data using the appropriate CFD-predicted fluxes (776.6 and 800.3 pmol/mm^2^/h/ppm, respectively). In the 2nd stage, a single value for *thick* was used in simultaneous optimization against all the DG and DPX adduct data. These stage 2 parameter values are the values shown in Table 2. All of these simulations assumed spatially uniform distribution of formaldehyde dehydrogenase activity.

#### Dose-response

**Figure 6.**
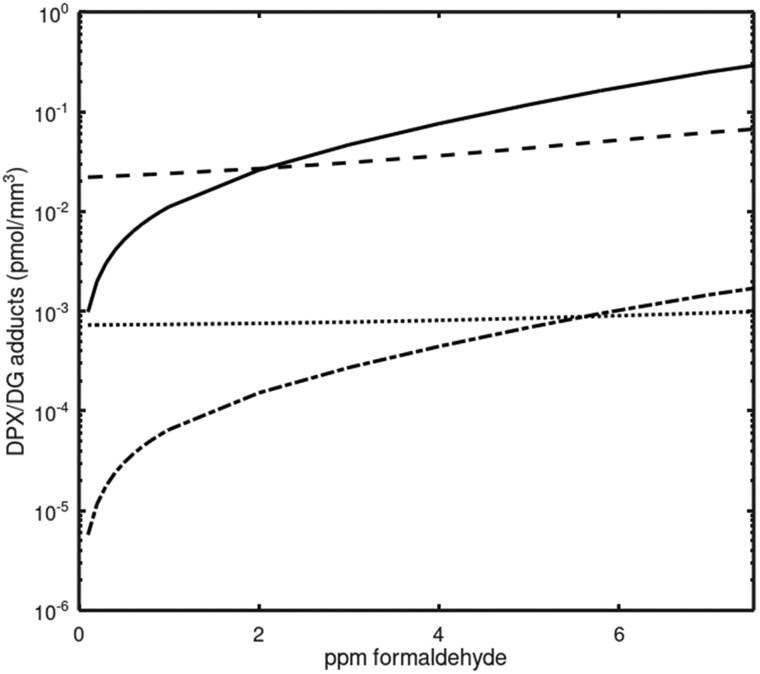
Simulated dose-response curves. Solid line and dashed line that crosses the solid line represent exogDPX and endoDPX, respectively. The concentration of exogDPX is predicted to cross that of endoDPX at about 2 ppm inhaled formaldehyde. Bottom 2 lines represent exogDG and endoDG. The concentration of exogDG is predicted to cross that for endoDG slightly below 6 ppm inhaled formaldehyde. Predicted concentrations of DPX adducts are over 10-fold higher than concentrations of DG adducts. Abbreviations: DG, deoxyguanosine; DPX, DNA-protein crosslink.

Dose-response curves were simulated for all 4 types of adducts (Figure 6). Below about 2 ppm inhaled formaldehyde, the concentrations of endogenous adducts exceeded concentrations of exogenous adducts. The dose-response curve for exogDPX is predicted to cross the curve for endoDPX at about 2 ppm inhaled formaldehyde, whereas the curve for exogDG is predicted to cross the curve for endoDG just below 6 ppm. Interestingly, the inhaled concentrations that have caused nasal tumors in rats, 6–15 ppm, are all concentrations at which the predicted concentrations of exogenous adducts exceed those of their endogenous counterparts.

## Discussion

###  

#### CFD modeling

A 3-dimensional, anatomically accurate CFD model was used to predict inhaled airflow and formaldehyde uptake in the rat nasal passages. The model reflects updated technologies for CFD analyses from prior efforts by Kimbell *et al*. (2001a,b). Notable updates include smoother surface geometry (Figure 1), refined computational mesh structure, and the use of updated numerical methods.

CFD calculations rely on a computational mesh of the surfaces and interior volumes of the model. Kimbell *et al*. (2001b) used hexahedral mesh structures derived from in-house software in the rat nasal model. Elements were designed to be as uniform in shape and size as possible but were relatively coarse by modern standards. Meshing technologies have increased dramatically in recent years allowing for high-quality, dense meshes of various structures to be developed in complex geometries. For this study, dense, unstructured tetrahedral meshes were developed that included a boundary layer of prism elements. Boundary layer meshes are recommended for mass transfer simulations to increase accuracy of calculations near the walls. Higher-quality meshes are likely responsible for the lower mass balance errors observed in this study. Kimbell *et al*. (2001b) reported errors for formaldehyde uptake as high as 14%. Those errors were accounted for by evenly distributing lost mass over the nasal surface and adjusting flux outputs accordingly. This step was not needed in the current work because of the small mass balance errors.

Despite differences in model geometries, computational mesh structures, and CFD software, predicted flux values from this study were like the results from Kimbell *et al*. (2001a,b) with a few exceptions. Flux predictions, including maximum and average values over the nonsquamous epithelium, were close between the 2 modeling approaches. Regional flux predictions displayed more differences between studies. For example, at the cell proliferation sites in the rat, flux predictions in this study were uniformly higher than those reported by Kimbell *et al*. (2001b). Differences in the anterior lateral meatus, posterior lateral meatus, anterior dorsal septum, and anterior medial septum regions were all <14% but differences in the posterior medial septum and medial maxilloturbinate regions were 28% and 34%, respectively.

#### Parameter values and DNA adduct data

A measured value of 3.024e9 base pairs was found for the Brown Norway rat genome (RGD, 2020) but no such value was found for F344 rats. The cell volume used for the conversion, 7.5e−7 mm^3^ (Bergstresser *et al*., 1978) was for human epidermal cells. No measurements of volume for rat nasal mucosal cells were found. Use of these values that are not from the F344 rat introduces some small uncertainty into the units conversion (Appendix 1), since variations in the genome are relatively minor between subspecies of animals (ie, <1%, including rats and humans and variations in cell volume). However, this uncertainty does not affect the qualitative dose-response and time-course characteristics of the adduct data and should not impinge on the model predictions of these data.

Although reasonably good descriptions were obtained of the adduct data (Figs. 4 and 5), there is always uncertainty in laboratory data and in the structure and parameterization of computational models that attempt to recapitulate the essential features of the systems that produce the data. The formation and disposition of 1-carbon fragments occurs in mitochondrial, cytosolic, and nuclear compartments and is not well-mixed across the volume of the cell (Tibbetts and Appling 2010; Field *et al*., 2018). Both Campbell *et al*. (2020) and the current work are thus incomplete descriptions of the intracellular compartmentalization of 1-carbon metabolism and of how this compartmentalization affects the formation of DG and DPX adducts. This uncertainty in model structure is relevant to the parameter values described n Table 2. For example, while the optimal value of kp, the zero-order rate of formation of endogenous formaldehyde, is 10 823 pmol/mm^3^/h, this composite value probably differs from the compartment-specific but currently unknown values (eg, mitochondrial, nuclear).

#### Model structure

**Figure 7.**
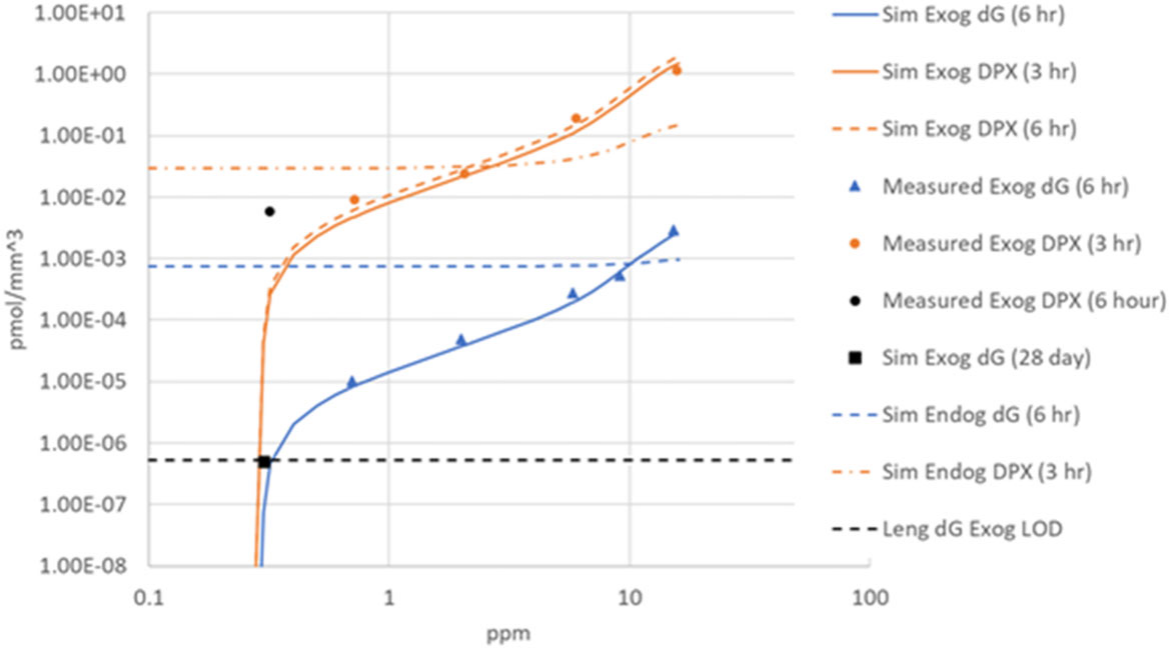
Dose-response data for exogenous DG adducts (blue triangle) (Lu *et al*., 2011) and for exogenous DPX (orange circle) (Casanova *et al*., 1994). The lower, black dashed line represents the limit of detection (LOD) for DG adducts (5.26e−7 pmol/mm^3^) (Leng *et al*., 2019). Casanova *et al*. (1994) data were obtained from mucosal tissue in the high tumor region of the rat nose after 3 hr of exposure. The DPX datum at 0.32 ppm (black circle; Casanova *et al*., 1989) was obtained from the combined respiratory and olfactory mucosal tissue after 6 h of exposure; therefore, this 0.32 ppm datum would have been somewhat higher in value if it had been sampled in the high tumor region but lower in value if it had been sampled at 3 rather than 6 h. The curves show the predictions of an alternative model structure that includes a saturable extracellular clearance of formaldehyde (Campbell *et al*., 2020) that was able to reproduce the nonlinear low-dose behavior of DG adduct formation suggested by Leng *et al*. (2019). The black square shows the model prediction with zero order extracellular clearance (2.02E + 03 pmol/mm^3^/h) which had been set to produce exogenous DG adducts under the Leng *et al*. (2019) exposure scenario (28 consecutive day exposure for 6 h/day to 0.3 ppm). The dosimetry model was not able to reproduce the low-dose behavior reported for both DPX and DG adducts using a single set of model parameters. Abbreviations: DG, deoxyguanosine; DPX, DNA-protein crosslink.

One of the striking results of simultaneously modeling DPX and DG adducts is the consistency of the dose-responses of these adducts as biomarkers of DNA exposure to formaldehyde. The model assumption that both DPX and DG are derived from the same source (cellular free formaldehyde) results in the successful prediction of the 2 experimental dose-responses at 0.7 ppm and above (Figure 4). However, there is an apparent disparity in the data at lower concentrations. Notably, Leng *et al*. (2019) did not find nasal mucosal exogDG adducts when F344 rats were exposed to 1, 30, or 300 ppb formaldehyde (ie, 0.001, 0.03, 0.3 ppm), 6 h/day, for 28 days, with a reported limit of detection (5.26e−7 pmol/mm^3^) several orders of magnitude lower than the adduct load observed at 2 ppm under the same experimental conditions (Figure 5). Together, the Leng *et al*. (2019) and Lu *et al*. (2011) results imply that a dosimetry threshold exists between 0.7 and 0.3 ppm, since Lu *et al*. (2011) found 1.026e−5 pmol exogDG adducts/mm^3^ after a single 6 h exposure at 0.7 ppm. Leng *et al*. (2019) suggest that the inherent reactivity of formaldehyde may result in a combination of extra- and intracellular clearance of exogenous formaldehyde that, at sufficiently low inhaled concentrations, effectively prevents exogenous formaldehyde from reaching nuclear DNA. In their analysis, Campbell *et al*. (2020) showed that a saturable extracellular clearance of inhaled formaldehyde could explain an adduct dose-response consistent with a threshold near 0.3 ppm. However, Casanova *et al*. (1989) had previously reported 5.74e−3 pmol exogDPX/mm^3^ in F344 rats exposed once to 0.32 ppm for 6 h. This positive result at 0.32 ppm is consistent with the exogDPX dose-response data at higher concentrations (Casanova *et al*., 1994) (Figure 7; Table 1). This low-dose discrepancy could be due to the higher specificity of the new method, because it only detects adducts at one of the bases in the DNA, whereas the older method detected DPX at all 4 bases. Perhaps the relative prevalence of formaldehyde adducts at the different DNA bases is not completely independent of formaldehyde concentration, and DG adducts are therefore relatively less frequent at low concentrations. Under this scenario, the Leng *et al*. (2019) hypothesis that combined extra- and intracellular clearance of inhaled formaldehyde can protect DNA could still be operative, but with an effective dosimetric threshold somewhat below 0.3 ppm.

Regardless of differences between the analytical techniques, the results of Casanova *et al*. (1994) and Leng *et al*. (2019) are largely consistent in demonstrating a highly non-linear response between 0.7 and 15 ppm with an approximate 126-fold increase in DPC and an approximate 286-fold increase in dG adducts, respectively, where the concentration of inhaled formaldehyde increased by approximately 22-fold (Table 1).

Given the potential significance for formaldehyde risk assessment of a nonlinear uptake process, we examined whether adding the saturable extracellular clearance description from Campbell *et al*. (2020) to the current model would make it possible to reproduce the disparate dose-responses. However, we were not able to reproduce the low-dose behavior reported for both DPX and DG adducts using a consistent set of parameters.

The prediction that levels of exogenous DG and DPX adducts only exceed the levels of their endogenous counterparts above 2–6 ppm inhaled formaldehyde (Figure 6) is informative, given that tumors were only seen in the inhalation bioassays at 6 ppm and above (Conolly *et al*., 2003). This dose-response behavior for the exogenous adducts, and for the total adduct burden, correlates with the dose-response for cytotoxicity that is an important determinant of the rat nasal tumor response (Conolly *et al*., 2002, 2003). Lastly, the current model has been updated to account for endogenous DPX related to formaldehyde. However, although formaldehyde is perhaps the most prevalent endogenous molecule resulting in DPX, DPX are formed by numerous endogenous processes: including, endogenous aldehylic DNA lesions crosslinking with proteins (eg, naïve apurinic/apyrimidinic AP sites and oxidized 2 deoxyriboses); reactive aldehydes crosslinking DNA and proteins; and proximal enzymes (eg, topoisomerases) transiently crosslinking to DNA, forming covalent DNA-enzyme intermediates (Nakamura and Nakamura 2020). The influence of total endogenous DPCs in relation to formaldehyde-specific DPCs has not been assessed in this model update.

CFD modeling supports prediction of flux from air into tissue at the specific anatomic sites where formaldehyde-related DPX and DG adducts were measured (Figure 2) and for the flux bins used in the BBDR modeling (Conolly *et al*., 2003; Kimbell *et al*., 2001a). However, little information exists on the spatial distribution of the biochemistry that affects adduct formation (Figure 3). Some information is available on regional differences in tissue thickness (Georgieva *et al*., 2003), but there are currently no datasets describing parameter values (Table 2) at the spatial resolution provided by CFD modeling. Thus, in the current modeling, the parameterization of the biochemistry that determines formation of DPX and DG adducts (Figure 3) is not site-specific. The same tissue thickness and biochemical parameter values are used throughout the nasal mucosa. In short, the high spatial resolution of the CFD modeling is not accompanied by correspondingly high spatial resolution of the relevant tissue biochemistry. The availability of such high spatial resolution tissue biochemistry data would be a welcome addition to computational modeling of the relationship between inhaled formaldehyde and adduct formation. However, in the absence of that data, this is the configuration of the tissue dosimetry model that will be used for a planned update of the rat BBDR model (Conolly *et al*., 2003).

## Supplementary Material

kfac119_Supplementary_Material

## Appendix 1

This Appendix describes how DG adduct data (Lu *et al*., 2011; Yu *et al*., 2015) with units of adducts/10^7^ deoxyguanosine and DPC data (Lai *et al*., 2016; Leng *et al*., 2019) with units of adducts/10^8^ deoxyguanosine were converted into units of pmol/mm^3^, the units of the DPX data (Casanova *et al*., 1989, 1994).

Input data

  41% of DNA base pairs contain deoxyguanosine (Piovesan *et al*., 2019)

    F_DG = 0.41

  The Norway rat has 3.024e9 base pairs per genome (RGD, 2020)

    num_bp = 3.024e9

  Avogadro’s number

    An = 6.02e23

  Volume of a cell (Bergstresser *et al*., 1978)

    Vcell = 7.5e−7  (mm^3^)

Units conversion. The following example is for the coversion of DG monoadduct data. For conversoin of the DPC crosslink data, 10^8^ DG should be used in place of 10^7^ DG.

    DG bp per genome

    DGbp = F_DG X num_bp

          = 1.2472e9

  DG adducts per genome (assume 1 DG adduct/10^7^ DG)

DG_adducts_per_genome  = DGbp/10^7^

               = 125

Molar concentration of DG adducts per cell    

 mol_DG_adducts_per_cell = DG_adducts_per_genome/An

                = 2.07e−22

Molar concentration of DG adducts per mm^3^

 mol_DG_adducts_per_mm^3^ = mol_DG_adducts_per_cell/Vcell

                = 2.632e−16

pmol DG adducts per mm^3^

 pmol_DG_adducts_per_mm^3^ = mol_DG_adducts_per_mm^3^/10^−12 ^pmol/mol

                  = 2.632e−4 pmol DG adducts/mm^3^

1 DG adduct/10^7^ DG = 2.632e−4 pmol DG adducts/mm^3^
