# Supplementary material for: Relative contributions of endogenous and exogenous formaldehyde to formation of deoxyguanosine monoadducts and DNA-protein crosslink adducts of DNA in rat nasal mucosa
Source: Toxicol Sci. 2022 Nov 21;191(1):15–24. doi: 10.1093/toxsci/kfac119 (PMC9887723; doi:10.1093/toxsci/kfac119)
Supplement: kfac119_Supplementary_Material [file kfac119_supplementary_material.docx]

**Relative contributions of endogenous and exogenous formaldehyde to formation of deoxyguanosine monoadducts and DNA-protein crosslink adducts of DNA in rat nasal mucosa**

**Supplemental Material**

These files together constitute the adduct dosimetry model for DPX and DG adducts. The code is written in Octave, which is a MATLAB clone. The code should run in MATLAB with only minor changes.

Run the model from Rat_dG_DPX_manual. This the only file you should have to interact with if you just want to run the model. Of course, the other files will be of interest if you want to examine the code. The differential equations are in the ode file.

**Rat_dG_DPX_data**

åç%dg and DPX adduct data for formaldehdye BBDR model

%Rory Conolly

%October 2, 2022

%Rat DPX data are from Casanova et al. (1994). TAP 23, 525-536.

%Sacrifice on the 5th day of the 12th week

%Exposures 6 hr/day, 5 days/wk

%Radiolabel exposures on the last day of the experiment were for 3 hr

%ppm DPX

Casa1989_data = [ 0.32 5.74e-3 ]; %(pmol/mm^3)

%ppm DPX

low_tumor_data = [ 0.72 1.64E-03

2.06 4.51E-03

6.01 3.81E-02

15.80 1.85E-01 ]; %(pmol/mm^3)

%ppm DPX

high_tumor_data = [ 0.72 9.02E-03

2.06 2.42E-02

6.01 1.94E-01

15.80 1.14 ]; %(pmol/mm^3)

%ppm time endog exog

Lu2011_data = [ 0.7 7 9.527E-04 1.026E-05

2.0 7 1.603E-03 5.001E-05

5.8 7 1.450E-03 2.737E-04

9.1 7 8.975E-04 5.343E-04

15.2 7 1.116E-03 2.935E-03 ]; %(pmol/mm^3)

%ppm time endog exog

Yu2015_data = [ 2.0 169 6.606E-04 9.212E-05

2.0 337 8.132E-04 2.211E-04

2.0 505 8.790E-04 2.500E-04

2.0 673 7.422E-04 2.763E-04

2.0 678 7.369E-04 2.184E-04

2.0 696 7.843E-04 2.106E-04

2.0 744 7.869E-04 1.658E-04

2.0 840 7.317E-04 1.763E-04 ]; %(pmol/mm^3)

**Rat_dG_DPX_engine**

%Formaldehyde dG & DPX adducts in rats

%Written in Octave

%Rory Conolly based on Campbell et al.

%October 2, 2022

%Read data from the m file "Rat_dG_DPX_params"

load_rat_flux_bins = 1;

load_rat_dG_DPX_params = 1;

load_rat_clonal_growth_params = 0;

load_rat_tumor_data = 0;

load_likelihood_data = 0;

Rat_dG_DPX_params;

load_rat_flux_bins = 0;

load_rat_dG_DPX_params = 0;

%Set case-specific metadata

if isequal(case_name, 'DPX_low')

concs = [ 0.1 0.2 0.3 0.4 0.5 0.6 0.7 0.8 0.9 1.0 2.0 3.0 4.0 5.0 ...

5.8 7.0 8.0 9.1 10.0 11.0 12.0 13.0 14.0 15. 15.8 17.0];

dG_DR_endo = zeros(length(concs),1);

dG_DR_exog = zeros(length(concs),1);

DPX_DR_endo = zeros(length(concs),1);

DPX_DR_exog = zeros(length(concs),1);

c.tstop = 168;

c.begin_inh = 0;

c.start_inh = 10;

c.stop_inh = c.tstop;

c.length_inh = 6;

c.wkend = 6;

c.thick = c.thick_low;

elseif isequal(case_name,'DPX_high')

concs = [ 0.1 0.2 0.3 0.4 0.5 0.6 0.7 0.8 0.9 1.0 2.0 3.0 4.0 5.0 ...

5.8 7.0 8.0 9.1 10.0 11.0 12.0 13.0 14.0 15. 15.8 17.0 ];

dG_DR_endo = zeros(length(concs),1);

dG_DR_exog = zeros(length(concs),1);

DPX_DR_endo = zeros(length(concs),1);

DPX_DR_exog = zeros(length(concs),1);

c.tstop = 168;

c.begin_inh = 0;

c.start_inh = 10;

c.stop_inh = c.tstop;

c.length_inh = 6;

c.wkend = 6;

c.thick = c.thick_high;

elseif isequal(case_name, 'Lu2011')

concs = [ 0.1 0.2 0.3 0.4 0.5 0.6 0.7 0.8 0.9 1.0 2.0 3.0 4.0 5.0 5.8 ...

7.0 8.0 9.1 10.0 11.0 12.0 13.0 14.0 15.2 16.0 17.0 ];

dG_DR_endo = zeros(length(concs),1);

dG_DR_exog = zeros(length(concs),1);

DPX_DR_endo = zeros(length(concs),1);

DPX_DR_exog = zeros(length(concs),1);

c.tstop = 18;

c.begin_inh = 0;

c.start_inh = 10;

c.stop_inh = c.tstop;

c.length_inh = 6;

c.wkend = 10;

c.thick = c.thick_swen;

elseif isequal(case_name, 'Yu2015')

concs = [ 2.0 ];

dG_time_course_endo = zeros(8,1);

dG_time_course_exog = zeros(8,1);

DPX_time_course_endo = zeros(8,1);

DPX_time_course_exog = zeros(8,1);

c.tstop = 850;

c.begin_inh = 0;

c.start_inh = 1;

c.stop_inh = 654;

c.length_inh = 6;

c.wkend = 10;

c.thick = c.thick_swen;

endif

C_F_air_bkgnd = 0.0;

if c.stop_inh > c.tstop

c.stop_inh = c.tstop;

endif

c.end_inh = c.start_inh + c.length_inh;

%LSODE is the algorithm that solves the differential equations

lsode_options("absolute tolerance",1e-3);

lsode_options("relative tolerance",1e-3);#Usually le-3

lsode_options("integration method","stiff");

lsode_options("maximum order", 5);

lsode_options("maximum step size", 0.01);

lsode_options("minimum step size", 1.e-20);

for zz = 1:length(concs) %run this loop for each concentration

%Schedule the discrete events - turning exposures on and off

C_F_air = concs(zz);

Rat_dG_DPX_scheduler;

simdatax = []; %Initialize

disp(' ')

disp(['Background ppm = ' num2str(C_F_air_bkgnd)])

disp(['Test ppm = ' num2str(C_F_air)])

%disp(['bin number ' num2str(i)])

c.fluxF = c.ratbins_flux ;%flux from air into tissue (pmol/hr/mm**2)

%Initial values for state variables

%1 Uptake

uptake_init = 0; %(pmol/mm^2)

%2 Endogenous acetal

C_endocyt_init = 20.; %(pmol/mm^3)

%3 Exogenous acetal

C_exogcyt_init = 0; %(pmol/mm^3)

%4 Glutathione

C_GSH_init = c.GSH_start; %(pmol/mm^3)

%5 Endogenous acetal bound to GSH

C_endocytsg_init = 120.; %(pmol/mm^3)

%6 Exogenous acetal bound to GSH

C_exogcytsg_init = 0; %(pmol/mm^3)

%7 Endogenous dG adducts

C_endodG_init = c.AenddG_start; %(pmol/mm^3)

%8 Exogenous dG adducts

C_exogdG_init = 0; %(pmol/mm^3)

%9 Endogenous DPX

C_DPX_endo_init = 0.01; %(pmol/mm^3)

%10 Exogenous DPX

C_DPX_exog_init = 0; %(pmol/mm^3)

%Vector of initial conditions for the odes

y0 = [ uptake_init

C_endocyt_init

C_exogcyt_init

C_GSH_init

C_endocytsg_init

C_exogcytsg_init

C_endodG_init

C_exogdG_init

C_DPX_endo_init

C_DPX_exog_init ];

%This loop solves the ODEs

for j = 1:length(events)-1

if events(j) == 0

tspan = [0 events(j+1)];

c.ci = param_values(1);

else

tspan = [events(j) events(j+1)];

if param_values(j) ~= -55555555.

c.ci = param_values(j);

endif

endif

%Run the odes. simdata holds the results

simdata = lsode('Rat_dG_DPX_ode',y0,tspan);

simdatax = [simdatax; simdata(end,:)];

%redefine the initial condition vector

y0 = simdata(end,:);

endfor %of loop for numerical integration from start to tstop

hours = 1:length(simdatax(:,1));

hours = hours*c.tstop/length(simdatax(:,1));

hours = hours(:);

%Prepare the simulated data for plotting

if isequal(case_name, 'DPX_low')

dG_DR_endo(zz) = simdatax(109,7);

dG_DR_exog(zz) = simdatax(109,8);

DPX_DR_endo(zz) = simdatax(109,9);

DPX_DR_exog(zz) = simdatax(109,10);

endif

if isequal(case_name, 'DPX_high')

dG_DR_endo(zz) = simdatax(109,7);

dG_DR_exog(zz) = simdatax(109,8);

DPX_DR_endo(zz) = simdatax(109,9);

DPX_DR_exog(zz) = simdatax(109,10);

endif

%Assume data collected 1 hr after end of exposure

if isequal(case_name, 'Lu2011')

dG_DR_endo(zz) = simdatax(17,7);

dG_DR_exog(zz) = simdatax(17,8);

DPX_DR_endo(zz) = simdatax(17,9);

DPX_DR_exog(zz) = simdatax(17,10);

endif

if isequal(case_name, 'Yu2015')

for ff = 1:length(Yu2015_data)

dG_time_course_endo = simdatax(:,7);

dG_time_course_exog = simdatax(:,8);

DPX_time_course_endo = simdatax(:,9);

DPX_time_course_exog = simdatax(:,10);

endfor

endif

endfor

%Return to Rat_dG_DPX_manual

**Rat_dG_DPX_manual**

%Script for manual running of the formaldehyde PK dG and DPX adduct model

%Rory Conolly

%October 2, 2022

%Ramboll formaldehyde BBDR model

clear all;

clc

close all;clc;format short e;more off;start_time=clock;

pkg load optim;pkg load statistics;

graphics_toolkit('qt'); % 'gnuplot' is also an option

%graphics_toolkit('gnuplot'); % 'qt' is also an option

t0 = clock;

clc

disp('Running dG_DPX_engine_for_rats')

%Miscellaneous

%global c p SSE opts costplot args Lu2011 Yu2015

global c p;

%===============================================================================

%Select dG or DPX adduct data to be analyzed

cases = { 'DPX_low' 0

'DPX_high' 0

'DPX_both' 0

'Lu2011' 0

'Yu2015' 0

'Yu_Lu' 0

'Lu2011_DPX_high' 1 }; %Run Lu & Yu together

%===============================================================================

%Inspect cases to find out which case is running

for i = 1:length(cases)

if cases{i,2} == 1

case_name = cases{i,1};

endif

endfor

%Now, set up the runs of "Rat_dG_DPX_engine" needed for the active case

if isequal(case_name, 'DPX_low')

Rat_dG_DPX_engine;

%Save for plotting

dG_DR_endo_low = dG_DR_endo;

dG_DR_exog_low = dG_DR_exog;

DPX_DR_endo_low = DPX_DR_endo;

DPX_DR_exog_low = DPX_DR_exog;;

%Plot results

close all

subplot(1,3,1), plot(hours,simdatax(:,10),'LineWidth',2)

hold on

grid on

subplot(1,3,1), plot(109,low_tumor_data(end,2),'MarkerSize',25)

axis([96,124])

title(['Low Tumor DPX, 15.8 ppm'])

xlabel('Time (hr)')

ylabel(['DPX (pmol/mm^3)'])

subplot(1,3,2), semilogy(concs, DPX_DR_exog,'--','LineWidth',2)

hold on

semilogy(low_tumor_data(:,1),low_tumor_data(:,2),'sm')

xlabel('ppm formaldehyde')

ylabel('DPX (pmol/mm^3)')

title('Low Tumor DPX')

subplot(1,3,3), semilogy(concs, DPX_DR_exog,'--','LineWidth',2)

hold on

subplot(1,3,3), semilogy(low_tumor_data(:,1),low_tumor_data(:,2),'sm')

subplot(1,3,3), semilogy(concs,DPX_DR_endo,'LineWidth',2)

subplot(1,3,3), semilogy(concs,dG_DR_endo,'LineWidth',2)

subplot(1,3,3), semilogy(concs,dG_DR_exog,'LineWidth',2)

xlabel('ppm formaldehyde')

ylabel('DPX (pmol/mm^3)')

elseif isequal(case_name, 'DPX_high')

Rat_dG_DPX_engine;

%Plot results

close all

subplot(1,3,1), plot(hours,simdatax(:,10),'LineWidth',2)

hold on

grid on

subplot(1,3,1), plot(109,high_tumor_data(end,2),'MarkerSize',25)

axis([96,124])

title(['High Tumor DPX, 15.8 ppm'])

xlabel('Time (hr)')

ylabel(['DPX (pmol/mm^3)'])

subplot(1,3,2), semilogy(concs, DPX_DR_exog,'--','LineWidth',2)

hold on

semilogy(high_tumor_data(:,1),high_tumor_data(:,2),'sm')

xlabel('ppm formaldehyde')

ylabel('DPX (pmol/mm^3)')

title('High Tumor DPX')

subplot(1,3,3), semilogy(concs, DPX_DR_exog,'--','LineWidth',2)

hold on

subplot(1,3,3), semilogy(high_tumor_data(:,1),high_tumor_data(:,2),'sm','LineWidth',2)

subplot(1,3,3), semilogy(concs,DPX_DR_endo,'LineWidth',2)

subplot(1,3,3), semilogy(concs,dG_DR_endo,concs,dG_DR_exog,'LineWidth',2)

xlabel('ppm formaldehyde')

ylabel('DPX (pmol/mm^3)')

%legend('Exogenous DPX','Exogenous DPX data','Endogenous DPX', ...

%'Endogenous dG','Exogenous dG', 'location','northeast')

figure

subplot(1,2,1), semilogy(concs, DPX_DR_exog,'k-','LineWidth',2)

xlim([0 18])

hold on

subplot(1,2,1),semilogy(concs,DPX_DR_endo,'k--','LineWidth',2)

subplot(1,2,1),semilogy(concs,dG_DR_endo,'k:','LineWidth',2)

subplot(1,2,1),semilogy(concs,dG_DR_exog,'k-.','LineWidth',2)

xlabel('ppm formaldehyde')

ylabel('DPX (pmol/mm^3)')

subplot(1,2,2), loglog(concs, DPX_DR_exog,'k-','LineWidth',2)

xlim([0.1 20])

hold on

subplot(1,2,2),loglog(concs,DPX_DR_endo,'k--','LineWidth',2)

subplot(1,2,2),loglog(concs,dG_DR_endo,'k:','LineWidth',2)

subplot(1,2,2),loglog(concs,dG_DR_exog,'k-.','LineWidth',2)

xlabel('ppm formaldehyde')

ylabel('DPX (pmol/mm^3)')

set(gca, "linewidth", 2, "fontsize", 18)

elseif isequal(case_name, 'DPX_both')

case_name = 'DPX_low';

Rat_dG_DPX_engine;

%Save for plotting

dG_DR_endo_low = dG_DR_endo;

dG_DR_exog_low = dG_DR_exog;

DPX_DR_endo_low = DPX_DR_endo;

DPX_DR_exog_low = DPX_DR_exog;;

case_name = 'DPX_high';

Rat_dG_DPX_engine;

%Save for plotting

dG_DR_endo_high = dG_DR_endo;

dG_DR_exog_high = dG_DR_exog;

DPX_DR_endo_high = DPX_DR_endo;

DPX_DR_exog_high = DPX_DR_exog;

subplot(3,2,1), plot(concs, DPX_DR_exog_low,'-','LineWidth',2)

hold on

subplot(3,2,1), plot(low_tumor_data(:,1),low_tumor_data(:,2),'*k')

xlabel('ppm formaldehyde')

subplot(3,2,1), plot(concs, DPX_DR_exog_high,'--','LineWidth',2)

subplot(3,2,1), plot(high_tumor_data(:,1),high_tumor_data(:,2),'sm')

xlabel('ppm formaldehyde')

ylabel('DPX (pmol/mm^3)')

title('Low & High Tumor Exoenous DPX')

subplot(3,2,2), loglog(concs, DPX_DR_exog_low,'-','LineWidth',2)

hold on

subplot(3,2,2), loglog(low_tumor_data(:,1),low_tumor_data(:,2),'*k')

subplot(3,2,2), loglog(concs, DPX_DR_exog_high,'--','LineWidth',2)

subplot(3,2,2), loglog(high_tumor_data(:,1),high_tumor_data(:,2),'sm')

xlabel('ppm formaldehyde')

ylabel('DPX (pmol/mm^3)')

title('Low & High Tumor Exogenous DPX')

subplot(3,2,3), loglog(concs, DPX_DR_endo_low,'LineWidth',2)

hold on

subplot(3,2,3), loglog(concs, DPX_DR_endo_high,'LineWidth',2)

xlabel('ppm formaldehyde')

ylabel('DPX (pmol/mm^3)')

title('Low & High Tumor Endogenous DPX')

subplot(3,2,4), loglog(concs, DPX_DR_exog_low,'-','LineWidth',2)

hold on

%subplot(3,2,4), loglog(low_tumor_data(:,1),low_tumor_data(:,2),'*k')

subplot(3,2,4), loglog(concs, DPX_DR_exog_high,'--','LineWidth',2)

%subplot(3,2,4), loglog(high_tumor_data(:,1),high_tumor_data(:,2),'sm')

subplot(3,2,4), loglog(concs, DPX_DR_endo_low,':','LineWidth',2)

subplot(3,2,4), loglog(concs, DPX_DR_endo_high,'-.','LineWidth',2)

xlabel('ppm formaldehyde')

xlabel('ppm formaldehyde')

ylabel('DPX (pmol/mm^3)')

title('Endogenous & Exogenous DPX')

subplot(3,2,5), loglog(concs, DPX_DR_endo_low,':','LineWidth',2)

hold on

subplot(3,2,5), loglog(concs, DPX_DR_endo_high,'-.','LineWidth',2)

subplot(3,2,5), loglog(concs, dG_DR_endo_low,'-','LineWidth',2)

subplot(3,2,5), loglog(concs, dG_DR_endo_high,'--','LineWidth',2)

xlabel('ppm formaldehyde')

xlabel('ppm formaldehyde')

ylabel('DPX (pmol/mm^3)')

title('Endogenous dG & Endogenous DPX')

subplot(3,2,6), loglog(concs, DPX_DR_exog_low,'-','LineWidth',2)

hold on

subplot(3,2,6), loglog(low_tumor_data(:,1),low_tumor_data(:,2),'*k')

subplot(3,2,6), loglog(concs, DPX_DR_exog_high,'--','LineWidth',2)

subplot(3,2,6), loglog(high_tumor_data(:,1),high_tumor_data(:,2),'sm')

subplot(3,2,6), loglog(concs, dG_DR_exog_low,'-','LineWidth',2)

subplot(3,2,6), loglog(concs, dG_DR_exog_high,'--','LineWidth',2)

xlabel('ppm formaldehyde')

xlabel('ppm formaldehyde')

ylabel('DPX (pmol/mm^3)')

title('Exogenous dG & Exogenous DPX')

case_name = 'DPX_both';

elseif isequal(case_name, 'Lu2011')

Rat_dG_DPX_engine;

%Plot results

figure

subplot(1,2,1), plot(concs,dG_DR_endo,'LineWidth',2)

hold on

subplot(1,2,1), plot(Lu2011_data(:,1),Lu2011_data(:,3),'db' )

subplot(1,2,1), plot(concs,dG_DR_exog','LineWidth',2)

subplot(1,2,1), plot(Lu2011_data(:,1),Lu2011_data(:,4),'xr')

xlabel('ppm')

ylabel('dG adducts')

title('Lu et al 2011 D-R')

subplot(1,2,2), loglog(concs,dG_DR_endo,'LineWidth',2)

hold on

subplot(1,2,2), loglog(Lu2011_data(:,1),Lu2011_data(:,3),'db' )

subplot(1,2,2), loglog(concs,dG_DR_exog','LineWidth',2)

subplot(1,2,2), loglog(Lu2011_data(:,1),Lu2011_data(:,4),'xr')

axis([0.1, 20, 1.e-6, 1.e-2])

xlabel('ppm')

ylabel('dG adducts')

title('Lu et al 2011 D-R')

elseif isequal(case_name, 'Lu2011_DPX_high')

case_name = 'Lu2011';

Rat_dG_DPX_engine;

figure

subplot(1,2,1), plot(concs,dG_DR_exog','k-.','LineWidth',2)

hold on

subplot(1,2,1), plot(Lu2011_data(:,1),Lu2011_data(:,4),'xk','MarkerSize',10)

xlabel('ppm')

ylabel('dG adducts (pmol/mm^3)')

%title('Lu et al 2011 D-R')

set(gca, "linewidth", 2, "fontsize", 18)

case_name = 'DPX_high';

Rat_dG_DPX_engine;

subplot(1,2,2), plot(concs, DPX_DR_exog,'k-','LineWidth',2)

hold on

subplot(1,2,2), plot(high_tumor_data(:,1),high_tumor_data(:,2),'sk','MarkerSize',10)

xlabel('ppm formaldehyde')

ylabel('DPX (pmol/mm^3)')

%title('High Tumor DPX')

set(gca, "linewidth", 2, "fontsize", 18)

elseif isequal(case_name, 'Yu2015')

Rat_dG_DPX_engine;

%Plot results

subplot(3,1,1), plot(hours, dG_time_course_endo)

hold on

subplot(3,1,1), plot(Yu2015_data(:,2), Yu2015_data(:,3),'xr')

title('Endogenous dG adducts')

xlabel('Hours')

ylabel('dG adducts')

subplot(3,1,2), plot(hours, dG_time_course_exog)

hold on

subplot(3,1,2), plot(Yu2015_data(:,2),Yu2015_data(:,4),'db')

title('Exogenous dG adducts')

xlabel('Hours')

ylabel('dG adducts')

subplot(3,1,3), plot(hours, dG_time_course_endo)

hold on

subplot(3,1,3), plot(Yu2015_data(:,2), Yu2015_data(:,3),'xr')

subplot(3,1,3), plot(hours, dG_time_course_exog)

subplot(3,1,3), plot(Yu2015_data(:,2),Yu2015_data(:,4),'db')

title('Exogenous & Exogenous dG adducts')

xlabel('Hours')

ylabel('dG adducts')

elseif isequal(case_name, 'Yu_Lu')

case_name = 'Lu2011';

Rat_dG_DPX_engine;

figure

plot(concs,dG_DR_endo,concs,dG_DR_exog)

hold on

plot(Lu2011_data(:,1),Lu2011_data(:,3),'db' )

plot(Lu2011_data(:,1),Lu2011_data(:,4),'xr')

xlabel('ppm')

ylabel('dG adducts')

title('Lu et al 2011 D-R')

%legend('Endogenous', 'Exogenous', 'location', 'northwest')

case_name = 'Yu2015';

Rat_dG_DPX_engine;

figure

subplot(3,1,1), plot(hours, dG_time_course_endo)

hold on

subplot(3,1,1), plot(Yu2015_data(:,2), Yu2015_data(:,3),'xr')

title('Endogenous dG adducts')

xlabel('Hours')

ylabel('dG adducts')

subplot(3,1,2), plot(hours, dG_time_course_exog)

hold on

subplot(3,1,2), plot(Yu2015_data(:,2),Yu2015_data(:,4),'db')

title('Exogenous dG adducts')

xlabel('Hours')

ylabel('dG adducts')

ylim([0 6.0e-4])

subplot(3,1,3), plot(hours, dG_time_course_endo)

hold on

subplot(3,1,3), plot(Yu2015_data(:,2), Yu2015_data(:,3),'xr')

subplot(3,1,3), plot(hours, dG_time_course_exog)

subplot(3,1,3), plot(Yu2015_data(:,2),Yu2015_data(:,4),'db')

ylim([0 1.e-3])

title('Exogenous & Exogenous dG adducts')

xlabel('Hours')

ylabel('dG adducts')

endif

%CPU time

y = etime(clock, t0);

CGminutes = y/60.;

disp(['Used ' num2str(CGminutes) ' minutes CPU time.'])

disp('Finished')

**Rat_dG_DPX_ode**

function integrate = Rat_dG_DPX_ode(statevalues, t)

%ODE section for Octave dG + DPX adduct model

%Rory Conolly

%October 2, 2022

global c

%Update the state variables for use with the ODEs

%2 Endogenous acetal

C_endocyt = statevalues(2);

%3 Exogenous acetal

C_exogcyt = statevalues(3);

%4 Glutathione

C_GSH = statevalues(4);

%5 Endogenous acetal bound to GSH

C_endocytsg = statevalues(5);

%6 Exogenous acetal bound to GSH

C_exogcytsg = statevalues(6);

%7 Endogenous dG adducts

C_endodG = statevalues(7);

%8 Exogenous dG adducts

C_exogdG = statevalues(8);

%9 Endogenous DPX

C_DPX_endo = statevalues(9);

%10 Exogenous DPX

C_DPX_exog = statevalues(10);

num_odes = 10; %Number of differential equations

integrate = zeros(num_odes,1); %initialize

%ODEs===========================================================================

%1 Uptake from inhaled air into cells lining the airway

uptake = c.fluxF*c.ci/c.thick; % (pmol/mm^3/hr)

integrate(1) = uptake; %(pmol/mm^3)

%2 Endogenous acetal

dC_endocyt = c.kp - c.k21*C_endocyt - c.k23*C_endocyt*C_GSH ...

+ c.k32*C_endocytsg - c.kDNA_dG*C_endocyt ...

- c.kDNA_DPX*C_endocyt + c.krep_dG*C_endodG; % (pmol/mm^3/hr)

integrate(2) = dC_endocyt; % (pmol/mm^3)

%3 Exogenous acetal

dC_exogcyt = uptake - c.k21*C_exogcyt - c.k23*C_exogcyt*C_GSH ...

+ c.k32*C_exogcytsg - c.kDNA_dG*C_exogcyt - c.kDNA_DPX*C_exogcyt ...

+ c.krep_dG * C_exogdG; %(pmol/mm^3/hr)

integrate(3) = dC_exogcyt; % pmol/mm^3)

%4 Glutathione

dC_GSH = c.k32*(C_exogcytsg+C_endocytsg) - c.k23*C_GSH*(C_exogcyt+C_endocyt) ...

+ c.Vmax*C_endocytsg/(c.Km*(1+(C_exogcytsg/c.Km)) + C_endocytsg) ...

+ c.Vmax*C_exogcytsg/(c.Km*(1+(C_endocytsg/c.Km))+ C_exogcytsg); %(pmol/mm^3/hr)

integrate(4) = dC_GSH; % (pmol/mm^3)

%5 Endogenous acetal bound to GSH

dC_endocytsg = c.k23*C_GSH*C_endocyt - c.k32*C_endocytsg ...

- c.Vmax*C_endocytsg/(c.Km*(1+(C_exogcytsg/c.Km)) + C_endocytsg);

% (pmol/mm^3/hr)

integrate(5) = dC_endocytsg; % (pmol/mm^3)

%6 Exogenous acetal bound to GSH

dC_exogcytsg = c.k23*C_GSH*C_exogcyt - c.k32*C_exogcytsg ...

- c.Vmax*C_exogcytsg/(c.Km*(1+(C_endocytsg/c.Km)) + C_exogcytsg);

% (pmol/mm^3/hr)

integrate(6) = dC_exogcytsg; % (pmol/mm^3)

%7 Endogenous dG adducts

dC_endodG = c.kDNA_dG*C_endocyt - c.krep_dG*C_endodG; % (pmol/mm^3/hr)

integrate(7) = dC_endodG; %(pmol/mm^3)

%8 Exogenous dG adducts

dC_exogdG = c.kDNA_dG*C_exogcyt - c.krep_dG*C_exogdG; % (pmol/m^3/hr)

integrate(8) = dC_exogdG; % (pmol/mm^3)

%9 Endogenous DPX

dC_DPX_endo = c.kDNA_DPX*C_endocyt - c.krep_DPX*C_DPX_endo;

% (pmol/m^3/hr)

integrate(9) = dC_DPX_endo ; % (pmol/mm^3)

%10 Exogenous DPX

dC_DPX_exog = c.kDNA_DPX*C_exogcyt - c.krep_DPX*C_DPX_exog; % (pmol/m^3/hr)

integrate(10) = dC_DPX_exog; % (pmol/mm^3)

%END

**Rat_dG_DPX_params**

%Rat_params

%October 2, 2022

%Load the adduct data

Rat_dG_DPX_data;

%These flux predictions by Jeff Schroeter, Nov. 10, 2020

if load_rat_flux_bins

if isequal(case_name, 'DPX_low')

c.ratbins_flux = 611.8;

elseif isequal(case_name, 'DPX_high')

c.ratbins_flux = 800.3;

elseif isequal(case_name, 'Lu2011') || isequal(case_name, 'Yu2015')

c.ratbins_flux = 776.6; %Swenberg sampling region

endif

end

%Rat formaldehyde dG adducts

if load_rat_dG_DPX_params

%Parameterization strategy: Use DPX values for Vmax, Km and k21.

%Use Campbell et al. for the other values.

%For optimization, focus on kp, k23, kDNA, krep, and thick

%Set to data or otherwise fixed

c.GSH_start = 4506.9801; %sets GSH_init (pmol/mm^3)

c.AenddG_start = 0.00074276; %sets AenddG_init (pmol/mm^3)

c.cint = 1.0;

c.k32 = 200;

%Optimized

c.kp = 10835.946; % zero-order production of endo formaldehyde (pmol/mm^3/hr)

c.k21 = 16.5774; % First-order loss of formaldehdye (1/hr)

c.k23 = 0.35; % 2nd-order binding of acetal with GSH (1/pmol/mm^3/hr)

c.krep_dG = 0.0063; % First-order loss of dG adducts (1/hr)

c.krep_DPX = 3.9e-1; % First-order loss of DPX adducts (1/hr)

c.kDNA_dG = 9.5183e-08; % First-order binding of acetal to dG (1/hr)

c.kDNA_DPX = 0.00018183; % First-order binding of acetal to DNA (1/hr)

c.Vmax = 74156; % Vmax for FDH (pmol/mm^3/hr)

c.Km = 1935.1847; % Km for FDH (pmol/mm^3)

c.thick_swen = 0.11388; % mucosal thickness (mm)

c.thick_high = 0.11388; % mucosal thickness (mm)

c.thick_low = 0.11388; % mucosal thickness (mm)

c.h = 2.0; % heteroskedasticity parameter

endif

% uptake_init = 0; %(pmol/mm^2)

% C_endocyt_init = 20.; %(pmol/mm^3)

% C_exogcyt_init = 0; %(pmol/mm^3)

% C_GSH_init = c.GSH_start; %(pmol/mm^3)

% C_endocytsg_init = 120.; %(pmol/mm^3)

% C_exogcytsg_init = 0; %(pmol/mm^3)

% C_endodG_init = c.AenddG_start; %(pmol/mm^3)

% C_exogdG_init = 0; %(pmol/mm^3)

% C_DPX_endo_init = 0.01; %(pmol/mm^3)

% C_DPX_exog_init = 0; %(pmol/mm^3)

**Rat_dG_DPX_scheduler**

%PROGRAM Rat_dG_scheduler

%Discrete event and ode scheduler

%Rory Conolly

%October 2, 2022

%Construct the event list, which defines the intervals over

% which the ODEs are solved. Parameter values may be changed

% at the start of each of these intervals. This allows,

% for example, for exposures to be started and stopped, with

% starting and stopping times specified in the hours of a

% 24-hr day for arbitrary numbers of days per week and of

% weeks. Also allowed are offsets from the start of the

% simulation (t=0) to the time in hours (not hour of the day)

% at which exposure starts. Similarly, exposures may end

% before the end of the experiment to simulate, for example,

% a recovery period.

global c

%disp('Scheduling...')

%Build arrays "starts" and "stops" that contain all possible

% starting and stopping times for exposures.

%Have to deal with any differences between (1) c.begin_inh and c.start_inh and

% (2) c.stop_inh and c.tstop

time = 0;

starts = [0];

stops = [];

while time < c.tstop

days = time/24 ;%day of experiment

weeks = days/7 ;%week of experiment

dow = (time - floor(weeks)*168.)/24. + 1. ;%day of week (1 - 7)

if dow < c.wkend

if time+c.start_inh < c.begin_inh

if time + c.end_inh > c.begin_inh

starts = [starts c.begin_inh];

if time+c.end_inh < c.stop_inh

stops = [stops time+c.end_inh];

else

stops = [stops c.stop_inh];

end

end

end

if time+c.start_inh >= c.begin_inh

if time+c.start_inh < c.stop_inh

starts = [starts time+c.start_inh];

if time+c.end_inh > c.stop_inh

stops = [stops c.stop_inh];

else

stops = [stops time+c.end_inh];

end

end

end

end

time = time + 24;

end

starts = unique(starts) ;%sorted array with unique values

stops = unique(stops) ;%sorted array with unique values

%Build the event array

events = [0:c.cint:c.tstop c.tstop];

events = union(events,starts) ;%sorted array with unique values

events = union(events,stops) ;%sorted array with unique values

%define cell array "param_names" to hold names of parameters whose

% values change at event times

param_names = {};

for i=1:length(events)

param_names{i} = 'null';

end

%define array to hold parameter values associated with events

param_values = -55555555.*ones(1,length(events));

%now populate the arrays

if c.begin_inh == 0 & c.start_inh == 0

param_names{1} = 'C_F_air';

param_values(1) = C_F_air_bkgnd + C_F_air;

else

param_names{1} = 'C_F_air';

param_values(1) = C_F_air_bkgnd;

end

for i = 2:length(starts)

param_names{(starts(i)+1)/c.cint} = 'C_F_air';

param_values((starts(i)+1)/c.cint) = C_F_air_bkgnd + C_F_air;

end

for i = 1:length(stops)

param_names{(stops(i)+1)/c.cint} = 'C_F_air';

param_values((stops(i)+1)/c.cint) = C_F_air_bkgnd;

end

%disp('Finished scheduling')
